# Photostabilized halocyanine for near‐infrared excited bimodal photothermal synergism and tumor‐targeted therapy

**DOI:** 10.1002/smo2.70088

**Published:** 2026-08-12

**Authors:** Zongjin Qu, Yurong Guo, Cuixia Yao, Can Wang, Zhen Wang, Jiali Li, Zhao Wang, Yingying Jing, Yen Leng Pak, Xing Gao, Shusheng Zhang

**Affiliations:** ^1^ Shandong Provincial Key Laboratory of Tumor Imaging Equipment Development and Diagnosis & Treatment Integration Technology Linyi University Linyi China; ^2^ College of Biological and Chemical Engineering Qilu Institute of Technology Jinan China; ^3^ College of Chemistry and Chemical Engineering Linyi University Linyi China

**Keywords:** halocyanine, photodynamic therapy, photostabilized, photothermal therapy

## Abstract

In this work, we replaced hydrogen with halogen atoms in the BC7 molecule to enhance the parent molecule's photostability and improve the photothermal therapy (PTT) and photodynamic therapy (PDT) effects. We further improve and enhance the properties of BC7 while maintaining its optical properties, tumor targeting, and optical therapeutic effects. Nuclear magnetic resonance (NMR) spectroscopy of the four synthesized compounds confirms the success of the target products. We briefly screen BC7‐Cl, BC7‐Br, and BC7‐I by in vitro photothermal experiments, in‐solution reactive oxygen species detection, and stability tests. We intravenously inject three molecules into a tumor‐bearing mouse model for in vivo tumor imaging and observe that their tumor‐targeting ability has been markedly improved. In further optical treatment experiments, BC7‐Br showed the best therapeutic effect and a significant improvement compared to BC7. This study provides innovative strategies and theoretical support for the construction and development of new materials with dual functions of efficient PTT and PDT.

## Graphical abstract

A series of halogenated organic small molecules were prepared by substituting hydrogen atoms in BC7 molecules with halogen atoms. These chemically modified derivatives displayed significantly improved stability, warming efficiency, and reactive oxygen species generation ability. BC7‐Br and BC7‐Cl showed remarkably enhanced therapeutic efficacy relative to BC7, with BC7‐Br exhibiting the optimal therapeutic effect, followed by BC7‐Cl.

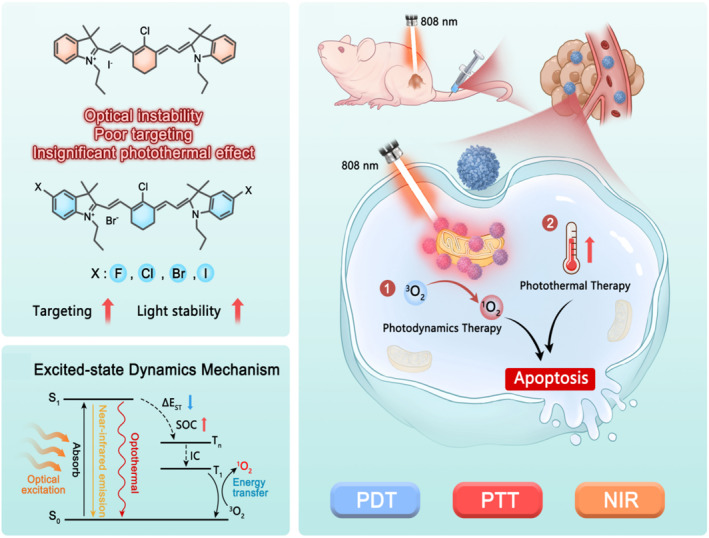

## 1 INTRODUCTION

Photodynamic therapy (PDT) achieves the killing of cancer cells through the singlet oxygen generated by photosensitizers under light activation.[^1^, ^2^, ^3^, ^4^, ^5^] During PDT treatment, oxygen consumption and the inherent hypoxic microenvironment of solid tumors may lead to insufficient oxygen supply, thereby affecting the efficacy of PDT.[^6^, ^7^, ^8^, ^9^] In contrast, photothermal therapy (PTT) is not limited by hypoxic environments and has advantages such as non‐invasiveness and low toxicity.[^10^, ^11^, ^12^, ^13^] Recent studies have shown that PDT and PTT can achieve dual‐mode phototherapy through their unique mechanisms, and their non‐overlapping toxicity characteristics make this combination therapy have significant potential therapeutic effects.[^14^, ^15^, ^16^, ^17^, ^18^]

However, the current implementation of combined phototherapy mainly relies on the use of a single functional component and different lasers, which makes the treatment process complex and time‐consuming.[^19^, ^20^, ^21^, ^22^] In addition, most reported photoactive agents (PAAs) with dual functions of PDT and PTT can only be activated by visible light, which inevitably limits the penetration depth of tissues and affects the therapeutic effect.[^23^, ^24^, ^25^, ^26^, ^27^] So far, some inorganic materials have been proven to possess PDT and PTT activity. However, the inherent defects of inorganic nanomaterials, such as poor biocompatibility, poor reproducibility of preparation processes, metabolic uncertainty, and long‐term safety issues, pose significant obstacles to clinical translation.[^28^, ^29^] Although organic nanomaterials have relatively superior safety, further improvement is still needed in terms of photostability.

As an ideal candidate material, through the design of cyanine dyes (BC7) of molecular structure, the regulation of excited state characteristics, the enhancement of targeting, and the development of new nano drugs, cyanine dyes have shown great potential in the treatment of tumors, inflammation, and the integration of diagnosis and treatment.[^30^, ^31^, ^32^, ^33^] Zhao's team developed the tumor targeting photosensitizer T780T by introducing tetraphenylethene with a distorted structure, forming nanoaggregates, and showed the tumor accumulation effect and significant tumor inhibition effect.^[34]^ Zhao's team optimized the excited state characteristics of pentamethine cyanine dyes, especially ANOMe‐Cy5, which improved the singlet oxygen yield and triplet lifetime, and effectively treated cancer cells.^[35]^ Li's team transformed anionic cyanine molecules into an efficient PTT reagent, C3T‐Pc, through anti‐ion engineering technology, which has red‐shift emission and enhanced singlet oxygen generation efficiency, and is suitable for tumor phototherapy.^[36]^ Wan's team transformed indocyanine green into a neutral cyanine drug mCy890, which has excellent photostability, photothermal conversion efficiency, and tumor targeting ability, and can effectively achieve tumor photothermal treatment.^[37]^ The Yue team has developed nitro‐modified cyanine Cy‐Mu‐7‐9 for the treatment of inflammatory bowel disease, which can be transformed into fluorescent probes to realize the integration of diagnosis and treatment.^[38]^

Research has found that chlorine‐containing dyes (such as 1‐Cl) can not only form non‐covalent complexes with serum albumin but also form covalent bonds. This type of dye enhances its targeting of tumors through a serum albumin‐mediated transport mechanism, rather than relying solely on the organic anion transport peptides pathway.[^39^, ^40^] However, due to its several drawbacks, such as poor water solubility, insufficient photostability under continuous near‐infrared laser irradiation, and the possibility of serious impact on targeting during its nanoscale process, PTT is not significantly effective. These factors greatly limit the further application of BC7 as a potential optical therapy drug. In addition, according to the energy gap law, the smaller the excited state energy level difference Δ*E*_ST_ of near‐infrared emitting materials, the faster the internal conversion (IC) rate, and the smaller the radiative transition and intersystem crossing (ISC). Therefore, an ideal PAA with dual functions of PDT and PTT needs to adjust its excited state energy distribution reasonably to achieve a subtle and balanced mechanism of near infrared (NIR) fluorescence, PDT, and PTT quantitatively evaluated.

**SCHEME 1:**
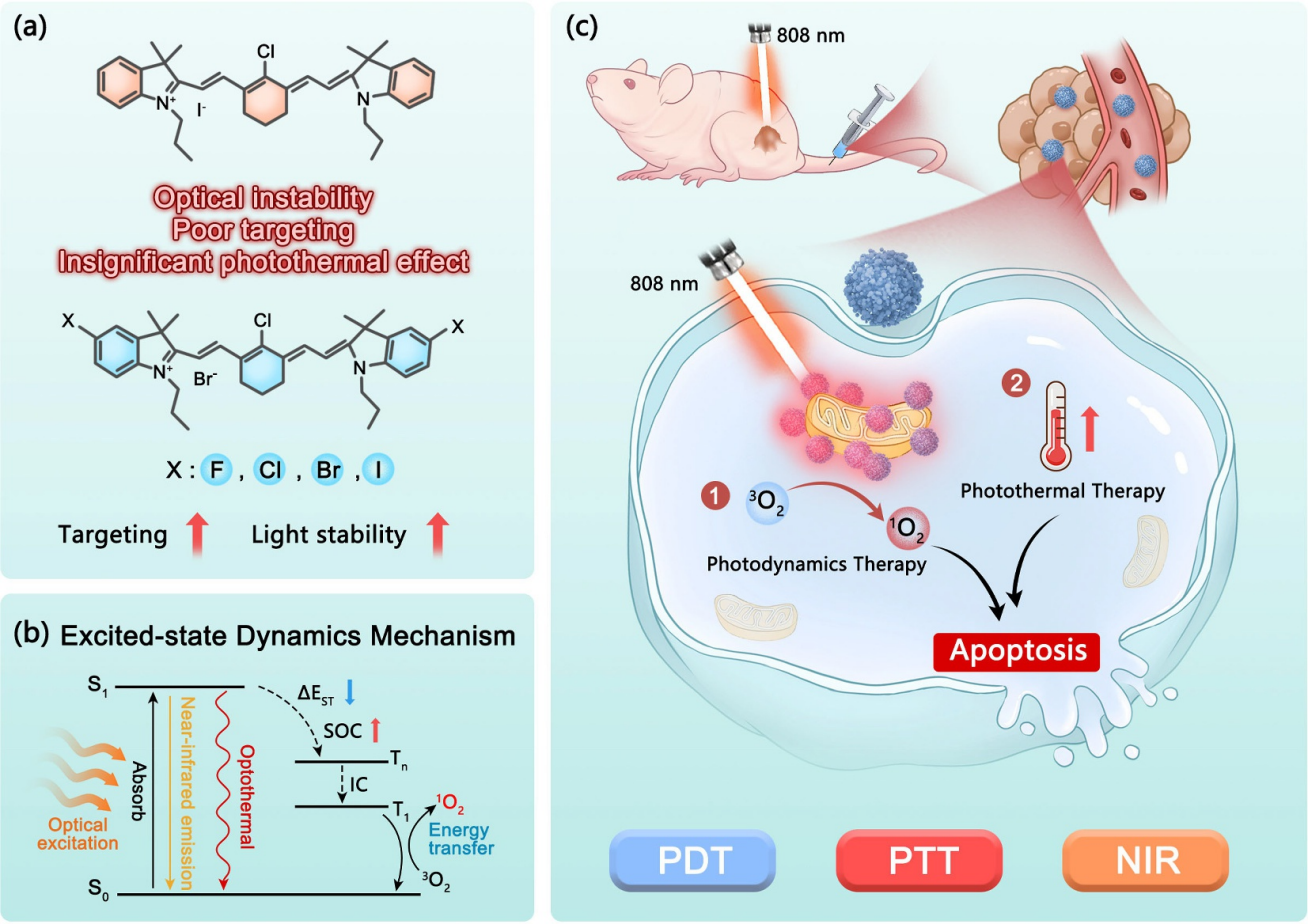
(a) Molecular structure of cyanine derivatives substituted with halogen atoms. (b) Description of the excited state dynamics mechanism of cyanine derivatives. (c) Schematic illustration of near‐infrared luminescence, photodynamic therapy, and photothermal therapy.

Herein, we attempted to develop a series of halogenated organic small molecules by replacing hydrogen atoms in BC7 molecules with halogen atoms (Supporting Information S1: Scheme S1). It is expected that these molecules can effectively inherit the tumor‐specific targeting properties of BC7 molecules, enhance their in vivo metabolic performance, and improve the efficacy of PDT (Scheme 1). We have successfully synthesized halogen‐substituted molecular derivatives of BC7‐F, BC7‐Cl, BC7‐Br, and BC7‐I. The four synthesized compounds were then subjected to nuclear magnetic resonance spectroscopy (NMR) analysis, and the results of spectral resolution confirmed the successful synthesis of the target products. Initial screening of the three molecules, BC7‐Cl, BC7‐Br, and BC7‐I, was conducted through in vitro photothermal experiments, in‐solution reactive oxygen species (ROS) detection experiments, and stability tests. These molecules were then administered to a tumor‐bearing mouse model via tail vein injection and subjected to in vivo tumor imaging experiments. Significant enhancement of the tumor‐targeting ability of the three molecules was observed. In subsequent PTT experiments, the results demonstrated that BC7‐Br exhibited the most optimal therapeutic effect, followed by BC7‐Cl. Both molecules exhibited a substantial therapeutic enhancement compared to BC7. Even BC7‐I, which has a relatively weak therapeutic effect, exhibited some performance improvement compared with BC7.

## 2 RESULTS AND DISCUSSION

### 2.1 Design and photophysical properties

**FIGURE 1:**
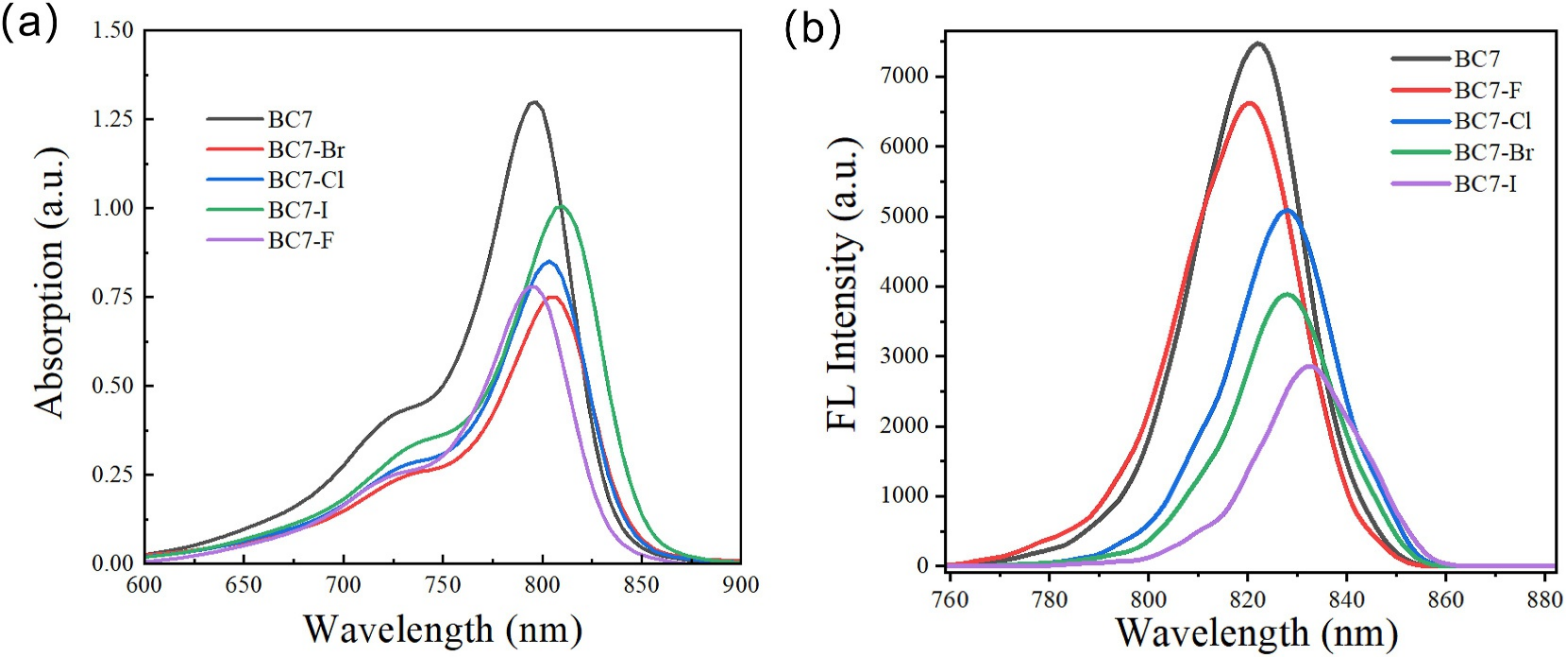
(a) Absorption spectra of DMSO solution with 5 μM concentration of five molecules; (b) emission spectra of DMSO solution with 10 μM concentration of five molecules. DMSO, dimethyl sulfoxide.

The molecular structures of the various halogenated derivatives were successfully determined by a combination of ^1^H‐NMR spectroscopy, ^13^C‐NMR spectroscopy, and electrospray ionization high‐resolution mass spectrometry (ESI‐HRMS) (Supporting Information S1: Figures S1–S14). To verify the near‐infrared developmental properties possessed by the BC7 molecule, the absorption and emission spectra of five molecules, namely, BC7‐F, BC7‐Cl, BC7‐Br, BC7‐I, and BC7, were examined in detail in dimethyl sulfoxide (DMSO) solvent. As shown in Figure 1, the maximum absorption (800 nm) and emission wavelengths (830 nm) of the molecular derivatives were located in the near‐infrared spectral region, indicating that they inherited the excellent near‐infrared fluorescence development properties of BC7 molecules. Therefore, these compounds have the potential to be used as fluorescent probes for optical imaging. In addition, it was found that with the enhancement of the heavy atom effect, both the absorption and emission wavelengths of the BC7‐X compounds showed a significant red‐shift phenomenon despite the decrease in the emission intensity of the compounds. This phenomenon may be closely related to the IC and ISC processes in the excited state. Thus, the lower fluorescence intensity of BC7‐I implies that it may dissipate more energy through the ISC pathway to transition to the *T*_1_ state.

**FIGURE 2:**
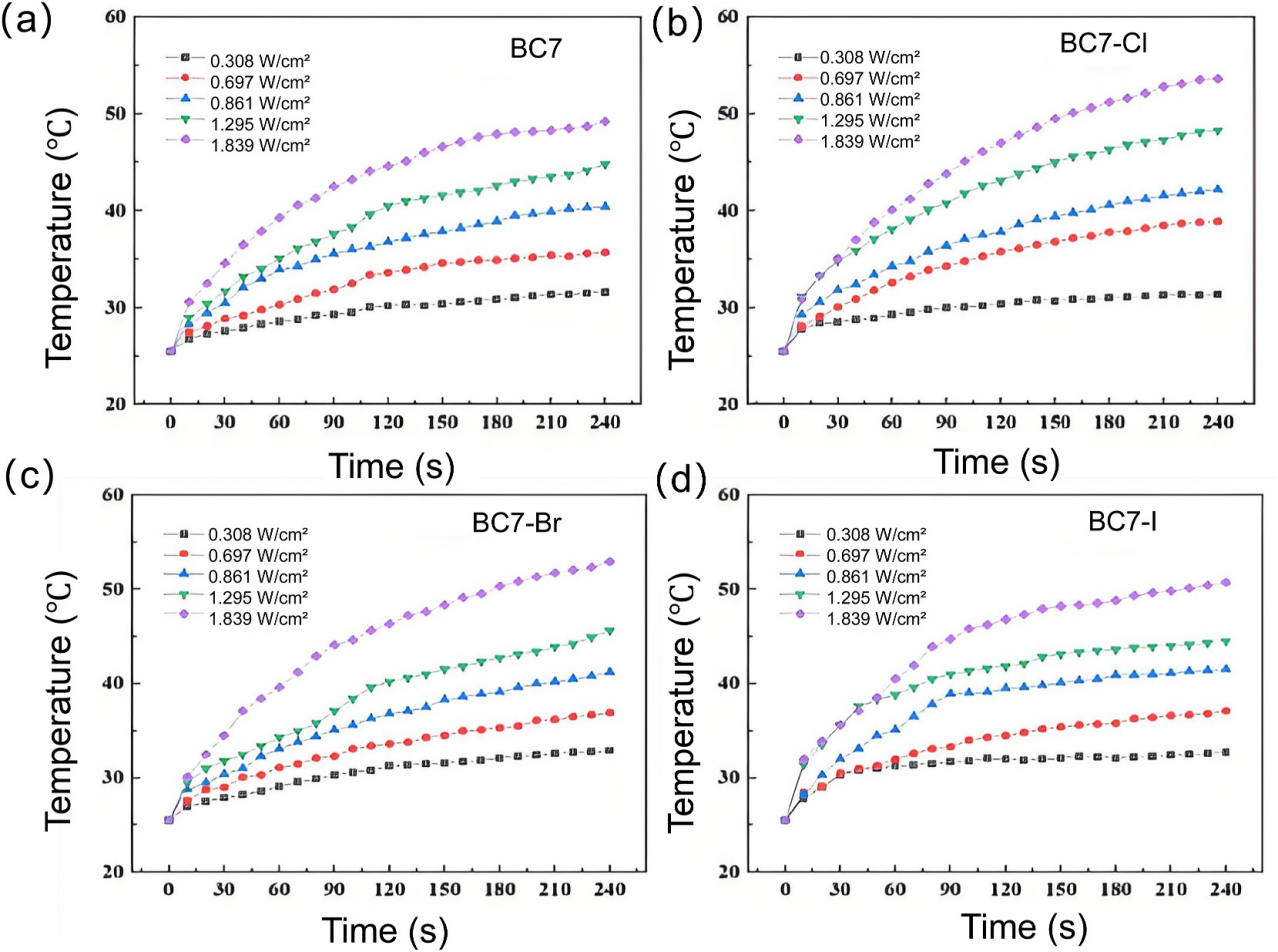
Temperature rise of various molecular compounds with different concentrations in DMSO solution under 808 nm laser irradiation (a) BC7, (b) BC7‐Cl, (c) BC7‐Br, (d) BC7‐I.

The photothermal effect is influenced by two key factors: laser power and drug concentration. In this study, we have fixed one of these factors to investigate the influence of the other on the in vitro temperature elevation effect. DMSO solutions of BC7‐Cl, BC7‐Br, BC7‐I, and BC7 with drug concentrations set at 8 μmol/L were subjected to temperature monitoring at varying power densities, and the corresponding temperature rise curves were plotted. The selected power density values were 0.308, 0.697, 0.861, 1.295, and 1.839 W/cm^2^, respectively. The irradiation was conducted using a near‐infrared laser for 4 min, and the temperature changes were recorded at an interval of once every 10 s. The experimental results are presented in Figure 2, where it can be observed that all the tested molecular solutions exhibited a significant temperature increase trend with increasing power density. Among them, the temperature increase from 25°C to 55°C was more significant for BC7‐Cl and BC7‐Br than for BC7, which increased from 25°C to 50°C. Notably, BC7‐I, despite its comparatively slower rate of warming, exhibited a photothermal effect that was analogous to that of BC7.

**FIGURE 3:**
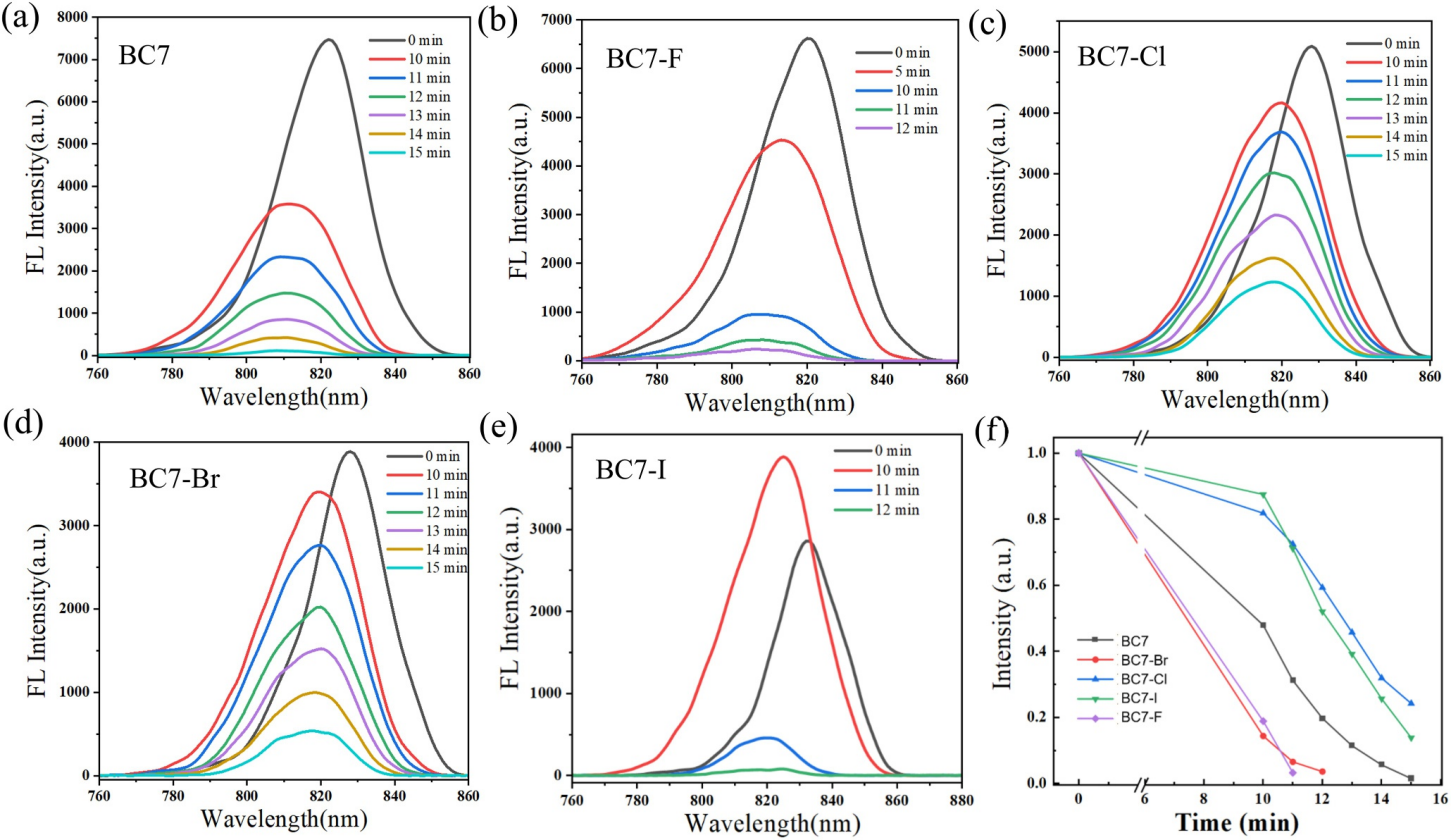
Fluorescence emission spectra of DMSO solutions of BC7‐Cl (a), BC7‐Br (b), BC7‐I (c), and BC7‐F (d) under 808 nm laser irradiation at different times. (e) Fluorescence emission spectra of the DMSO solution of BC7 under 808 nm laser irradiation at different times. (f) Fluorescence emission ratio of BC7‐F/BC7‐Cl/BC7‐Br/BC7‐I and BC7 under different lighting times. DMSO, dimethyl sulfoxide.

To deeply investigate the differences in photostability under NIR irradiation between BC7‐F, BC7‐Cl, BC7‐Br, BC7‐I, and BC7, we performed 808 nm laser irradiation experiments on the above molecules in DMSO solution at 10 μmol/L concentration. Fluorescence tests were performed on the treated solutions under the same experimental conditions to evaluate the effect of laser irradiation on the fluorescence emission of each solution. The irradiation condition with a power density of 0.861 W/cm^2^ was determined by repeating the experiment several times. Figure 3 shows that the fluorescence emission of BC7 decreased significantly with the increase in light exposure time, and the fluorescence emission was almost zero after 15 min of irradiation. Among the four halogen‐derived molecules, BC7‐Cl and BC7‐Br remained weakly fluorescent after 15 min, showing that their photostability under NIR irradiation was significantly better than that of BC7 and was significantly enhanced. However, BC7‐F and BC7‐I were slightly less stable, and the fluorescence was almost zero after 12 min, which is consistent with previous reports.^[30]^ Figure 3f demonstrates the ratio of fluorescence emission data of different molecular compound solutions after different light exposure times, thus reflecting more intuitively the superior stability of BC7‐Cl and BC7‐Br compared to BC7. The fluorescence attenuation observed in the experiment only reflects the fluorescence retention situation and is not a direct measurement of the integrity of the structure. According to the photothermal conversion efficiency formula of photosensitizers (Supporting Information S1: Figure S15), we obtained that the photothermal conversion efficiency of different photosensitizers were 30.1% for BC7, 36.7% for BC7‐Cl, 34.2% for BC7‐Br and 31.5% for BC7‐I.

**FIGURE 4:**
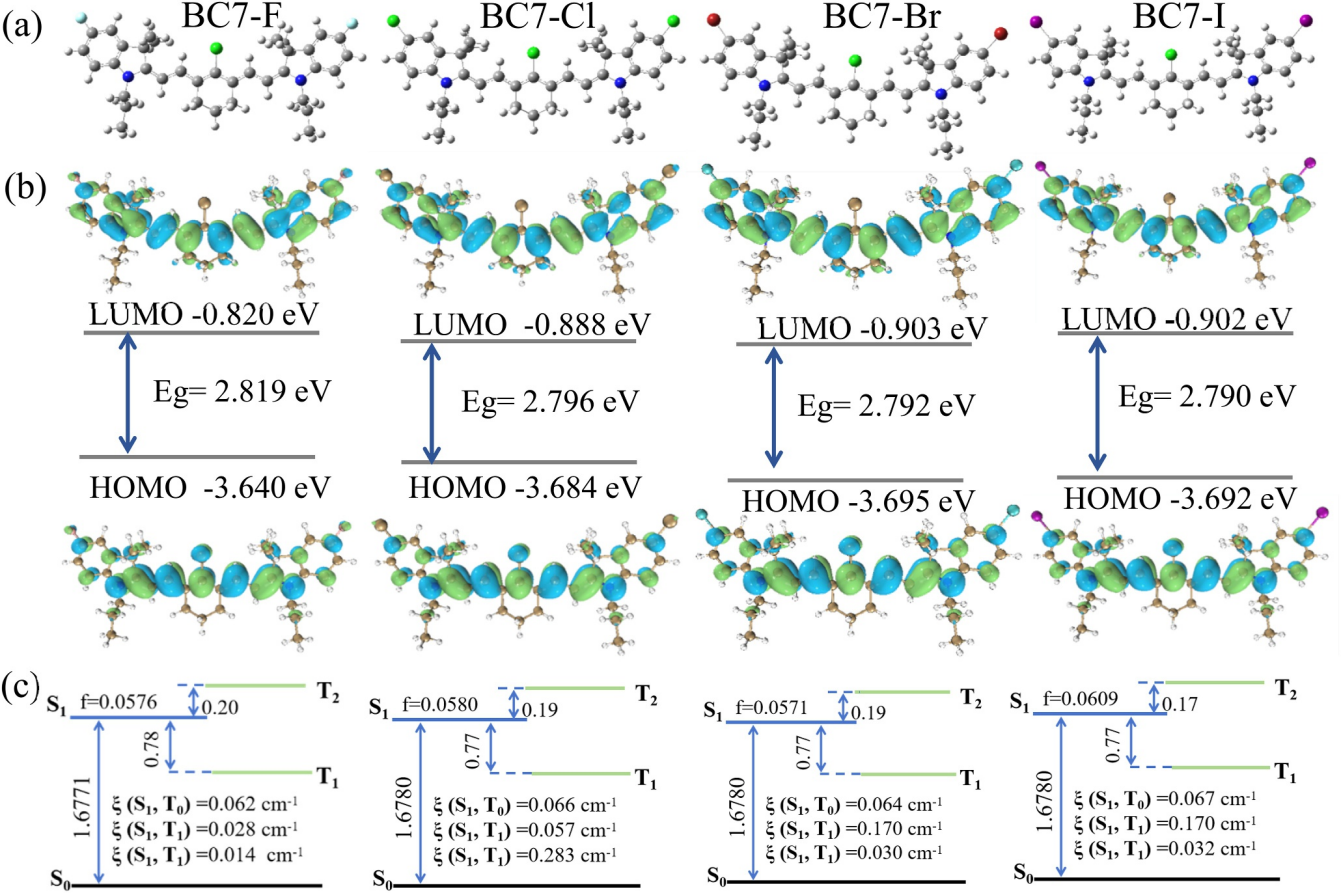
Theoretical calculation analysis (a) Gaussian optimization of the most stable structure of BC7‐X compounds. (b) HOMO/LUMO surface of geometry‐optimized BC7‐X in DMSO. (c) Calculated energy levels of *S*_1_ and the lowest three *T*_*n*_ states, and the corresponding oscillator strength *f*, energy band gaps between *S*_1_ and *T*_*n*_, and SOC matrix elements for BC7‐X. HOMO, highest occupied molecular orbital; LUMO, lowest unoccupied molecular orbital; SOC, spin‐orbit coupling.

### 2.2 Theoretical calculations

To further explain the experimental results, density‐functional theory (DFT) and time‐dependent density‐functional theory (TD‐DFT) calculations were performed using the B3LYP generalization and the def2svp motifs from the Gaussian16 package.^[41]^ BC7‐X exhibits similar optimal ground‐state geometries, which are attributed to their shared molecular backbone structures, which show nearly coplanar arrangements and significant conjugation effects (Figure 4a and Supporting Information S1: Figure S16). Further analysis showed that the *S*_0_ to *S*_1_ transitions of all examined compounds were mainly controlled by the highest occupied molecular orbital (HOMO) to lowest unoccupied molecular orbital (LUMO) transitions (Figure 4b). Although the substitution of halogen atoms did not significantly affect the HOMO/LUMO distribution, its modulating effect on the energy levels was significant.

Specifically, halogen‐substituted compounds exhibit reduced HOMO and LUMO energy levels due to the electron attraction effect of the halogen atoms, resulting in a decrease in the HOMO/LUMO energy level difference. The slight enhancement of the HOMO and LUMO energy levels of BC7‐I compared to BC7‐Br can be attributed to its larger electron cloud size, which makes the outermost lone pair of electrons more susceptible to conjugate interactions with neighboring π‐electron clouds, thus weakening the electron attraction effect. In addition, HOMO is mainly localized near the negatively charged central bromine atom, whereas LUMO is distributed throughout the molecular backbone. Therefore, the role of halogen atoms is more significant in HOMO compared to LUMO. The proper atomic overlap between HOMO/LUMO usually ensures a sufficiently large vibronic strength (*f*), which supports the strong light absorption of BC7‐X. The spectral profiles of BC7‐X in the free state from time‐dependent TD‐DFT calculations are in high agreement with the experimental data in terms of peak shape and full‐width‐half‐maximum (FWHM), which verifies the reliability of the adopted calculation method (Supporting Information S1: Figure S17).

In the ISC channel between the *S*_1_ and *T*_2_ energy levels, it is observed that the smaller energy level difference between *S*_1_ and *T*_2_ contributes to the efficient ISC (Figure 4c), with values ranging from 0 to 0.17 eV. Further, we evaluate the spin‐orbit coupling (SOC) matrix element (*ξ*) for the *S*_1_ and *T*_2_, *T*_3_, and *T*_4_ channels and found that the SOC was significantly enhanced in the *S*_1_ − *T*_2_ channels. The SOCs of BC7‐Cl, BC7‐Br, and BC7‐I exhibited a significant increase compared to BC7 (Supporting Information S1: Figure S18) and BC7‐F, which was attributed to the overlap of the electron cloud between the halogen atoms and the chromophores, leading to the enhancement of inter‐electronic interactions, which in turn facilitated spin‐orbital coupling. The SOC value of BC7‐I was several times higher than that of BC7, a result that verified that SOCs were efficiently enhanced by iodination treatment. Therefore, the increase of *ξ*(*S*_1_ − *T*_1_) together with the decrease of $\Delta E_{\left(S_{1}-T_{1}\right)}$ promotes the enhancement of the ISC efficiency of BC7‐I concerning BC7, which in turn improves the Φ^1^O_2_ observed in the experiment.

### 2.3 Evaluation of reactive oxygen species generation in solution

1,3‐Diphenylisobenzopyran (DPBF), as a fluorescent/colorimetric probe specific for singlet oxygen, possesses the ability to detect the generation of ROS. 5 μmol/L of the molecule to be tested was mixed with 200 μmol/L of DPBF solution in a 1:1 ratio (both with DMSO as the solvent), and the absorbance changes at different time points under 808 nm laser irradiation were determined. Three different power densities, that is, 0.308, 0.861, and 1.295 W/cm^2^, were selected for the experiments, and the UV absorbance measurements were performed every 30 s. At 0.308 W/cm^2^ power density, the absorbance of the DMSO solution of DPBF at 415 nm wavelength remained stable with the light exposure time, which confirms that DPBF itself does not undergo photochemical changes under sustained light exposure conditions.[^42^, ^43^]

**FIGURE 5:**
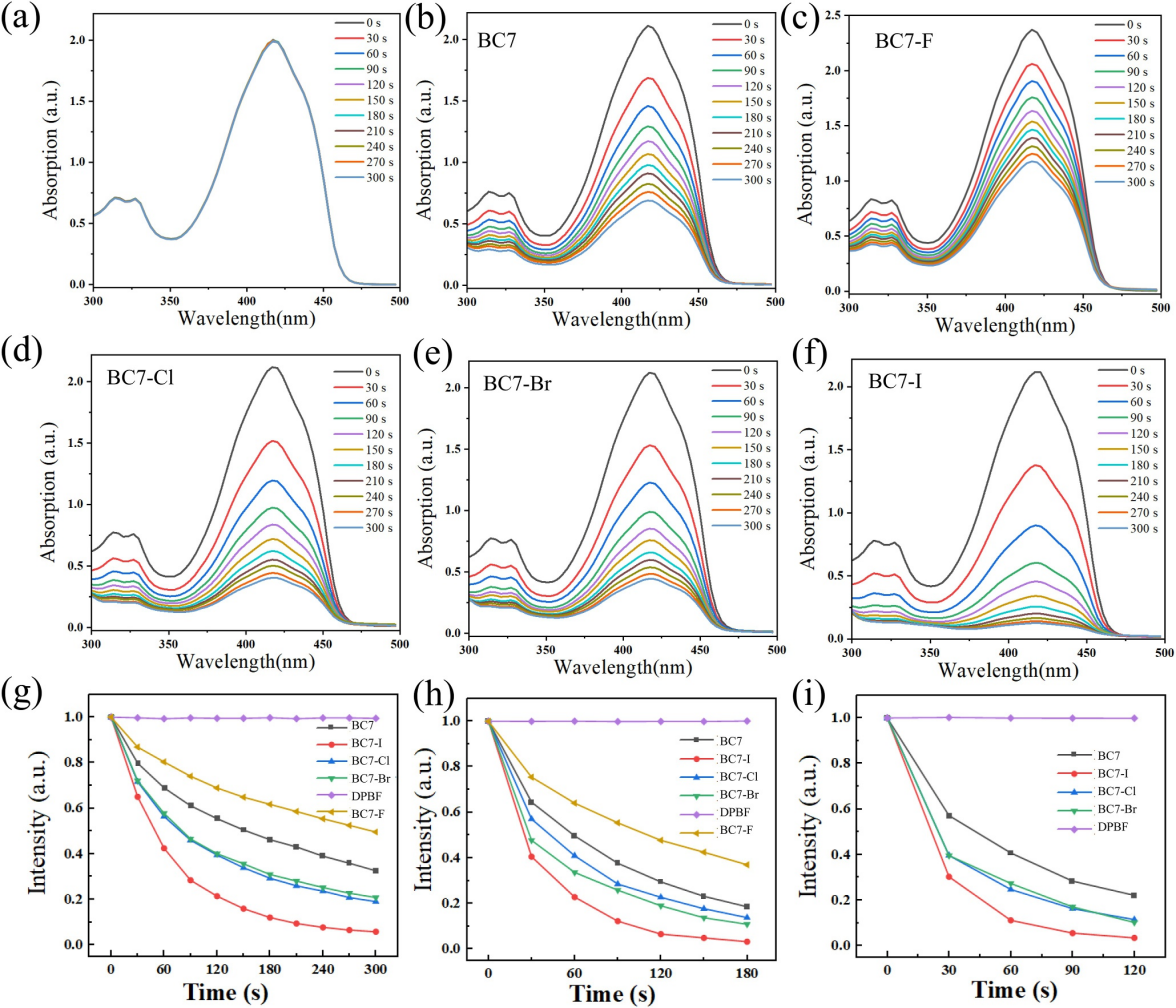
Absorption spectra of (a) DPBF solution and DMSO mixed solution of DPBF and (b) BC7, (c) BC7‐F, (d) BC7‐Cl, (e) BC7‐Br, (f) BC7‐I under 808 nm laser irradiation with a power density of 0.308 W/cm^2^. The absorbance ratios of DMSO mixed solutions of various molecules and DPBF under different power densities of 808 nm laser irradiation: (g) 0.308 W/cm^2^, (h) 0.861 W/cm^2^, (i) 1.295 W/cm^2^. DMSO, dimethyl sulfoxide; DPBF, 1,3‐diphenylisobenzopyran.

Figure 5b–f show the variation of absorbance with exposure time after mixing 200 μmol/L concentration of DPBF with 5 μmol/L concentration of DMSO solutions of BC7, BC7‐Cl, BC7‐F, BC7‐Br, and BC7‐I, respectively. The absorbance of all mixed solutions showed a decreasing trend with the prolongation of light irradiation time. This indicates that these molecules possess the ability to generate ROS under laser irradiation, including BC7. BC7‐I has a particularly prominent ROS generation ability, while BC7‐Cl and BC7‐Br also have significantly better ROS generation ability compared to BC7. Figure 5g–i show the absorbance ratios of the mixed solutions concerning the illumination time at three different power densities, which more intuitively reflect the advantages of BC7‐Cl, BC7‐Br, and BC7‐I in ROS generation compared to BC7 output.

### 2.4 Cytotoxicity and reactive oxygen species assays

**FIGURE 6:**
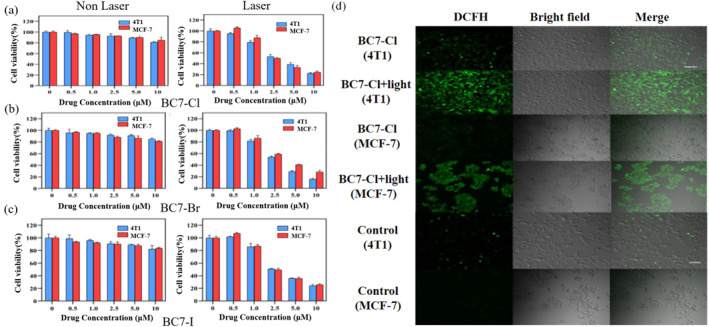
Cytotoxicity tests of different concentrations of BC7‐Cl, BC7‐Br, and BC7‐I on 4T1 and MCF‐7 cells: (a) non‐laser and laser irradiation cytotoxicity of BC7‐Cl. (b) Non‐laser and laser irradiation cytotoxicity of BC7‐Br. (c) Non‐laser and laser irradiation cytotoxicity of BC7‐I. (d) DCFH was used as a probe to detect reactive oxygen production in 4T1 and MCF‐7 cells (bar = 50 μm). DCFH, dichlorofluorescein.

The cytotoxicity (both dark and phototoxic) of several molecular solutions at different concentrations on 4T1 cells and MCF‐7 cells was systematically evaluated by the cell counting kit‐8 method. Figure 6a–c demonstrate the experimental data for the group to which the laser was applied, and the results show a significant decrease in the survival of tumor cells as the concentration of the drug increased, thus confirming the presence of a tumor treatment effect. In addition, the same light treatment was applied to normal cells, as shown in Supporting Information S1: Figure S19, the cell survival rate decreased after light exposure, but the difference was not significant, which verified the safety of the synthesized molecules. Further findings support the photothermal and photodynamic therapeutic effects of the series of molecules with significant inhibitory effects on tumor cell growth under light conditions.

In order to precisely investigate the effects of different drug molecules in inducing ROS generation in cells, we used dichlorofluorescein diacetate (DCFH‐DA) as an indicator of ROS and observed it under a confocal microscope. The intensity of green fluorescence was used as a quantitative indicator of ROS generation, and the enhancement of its intensity directly reflected the increase in ROS generation. The experimental results showed that in Figure 6d, the experimental group with applied laser irradiation showed a significant increase in green fluorescence generation compared with the control group without applied laser, which revealed that the light condition could significantly induce a large amount of ROS generation. In addition, the control group with only light applied and no drug molecules added observed extremely low and almost negligible fluorescence intensity, which indicates that ROS are not spontaneously generated in the cells in the absence of drug molecules.

### 2.5 Tumor in vivo imaging and therapy

**FIGURE 7:**
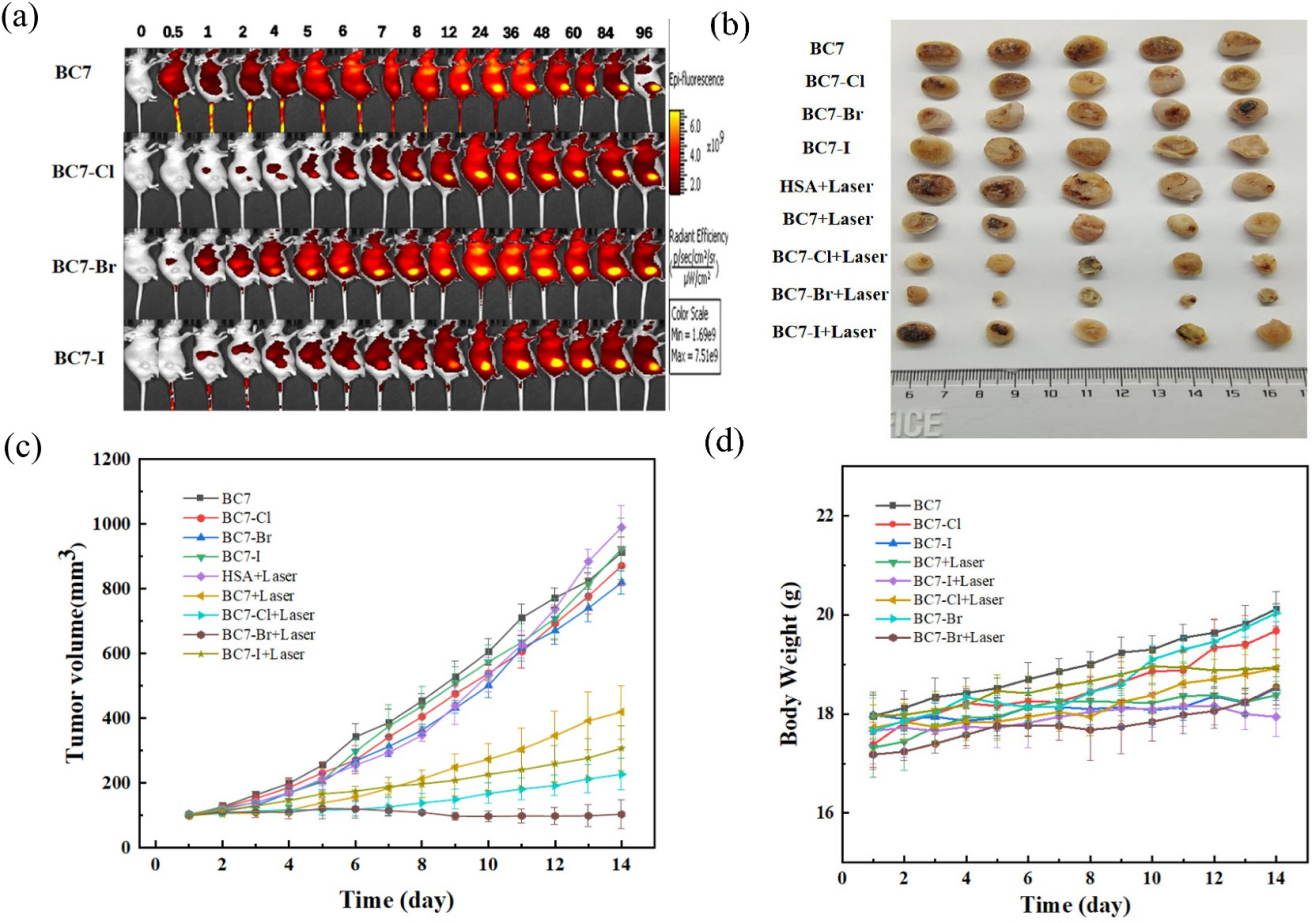
(a) Comparison of fluorescence imaging between BC7 and BC7‐Cl, BC7‐Br, BC7‐I on tumor‐bearing mice under the same experimental conditions. (b) In vitro tumor photos after the end of the treatment cycle. (c) The corresponding tumor volume change curves of each group of mice during the treatment process. (d) The corresponding weight change curves of mice in each experimental group during the treatment process.

We explored their distributional properties in vivo in a nude mouse loaded tumor model. Based on previous experimental studies, human serum albumin (HSA) possesses the ability to bind to a wide range of organic small‐molecule compounds and to shield its hydrophobicity, thereby enhancing its solubility in plasma. Combined with the actual situation of current clinical treatment, hypoproteinemia often occurs after major surgery, chronic diseases, massive blood loss, burns, etc., at which time it is usually necessary to supplement HSA by intravenous injection. Therefore, HSA was chosen as a cosolvent to formulate the intravenous injection of each molecule. We selected several rhabdomyosarcoma mice without trauma and with appropriate tumor growth status, and the mother liquids of BC7, BC7‐Cl, BC7‐Br, and BC7‐I were diluted with phosphate buffered saline (pH = 7.4) containing 20 mg/mL of HSA to prepare a solution for injection and injected into rhabdomyosarcoma mice by tail vein injection (100 μL each). Immediately after injection, the mice were anesthetized, and fluorescence in vivo imaging was performed; the anesthesia was removed after imaging, and reanesthetized before the next imaging. In vivo imaging of each derivative molecular compound was performed at different time points after injection using a fluorescence in vivo imaging system, and as shown in Figure 7a, BC7‐Br and BC7‐Cl molecules accumulated more rapidly in living tumors than BC7.

PDT and PTT, as two emerging tumor treatment modalities in the field of optical therapy, both rely on the irradiation of laser light at specific wavelengths to stimulate the optical therapeutic agents to produce ROS and/or heat, and to achieve tumor treatment. As the core of this therapeutic modality, the performance of optical therapeutics is directly related to the therapeutic effect. We observed through in vitro warming experiments that this series of derivative molecules demonstrated an excellent in vitro warming effect under 808 nm laser irradiation. Meanwhile, the absorbance curve of the mixed solution with DPBF decreased continuously with the prolongation of light exposure time, revealing its efficient ROS generation ability. In addition, the in vitro tumor cell killing assay under NIR illumination also confirmed its efficient cytotoxicity. The in vivo targeting experiments also confirmed the excellent performance of BC7‐Cl and BC7‐Br molecules in tumor targeting. A series of experimental results indicates that these derivative molecules are capable of exerting dual therapeutic effects of both PTT and PDT.

Based on this, we further explored the optical therapeutic effects of these derivative molecules in a hormonal mouse model. As shown in Figure 7b, after the treatment cycle, ex vivo images of tumor anatomy revealed that under 808 nm laser irradiation, the tumor volume of the experimental group injected via tail vein was significantly reduced compared to that of the group with no light applied as well as that of the group containing only HSA with light applied, and that the tumor volume of the BC7‐Br + laser group was significantly smaller than that of the BC7 + laser group compared to the BC7‐Cl + laser group, indicating a significant enhancement of the tumor therapeutic effect compared to the BC7 + laser group, which indicated a significant enhancement of the tumor treatment effect compared to BC7.

During the optical treatment experiments, precise quantitative measurements of tumor volume were made at established time points, and the body weights of the experimental mice were continuously monitored. Subsequently, the collected data were graphed. As shown in Figure 7c, the tumor volumes of the four groups of experimental mice, not treated with light, as well as the control group to which HSA light was applied, did not exhibit a significant therapeutic effect. In contrast, the tumor volume growth rate of the four experimental mice in the BC7 + Laser group, BC7‐Cl + Laser group, BC7‐Br + Laser group and BC7‐I + Laser group was significantly reduced, and some of the experimental mice even showed a reduction in the tumor volume, a result that fully confirms the significant application of the synthesized halogen derivative molecule in the field of optical therapeutic potential. As can be observed in Figure 7d, the weight changes of the nine groups of experimental mice showed a gradual increase in body weight, and the normal growth of the mice was not significantly affected whether or not light was applied during the treatment period, thus verifying the drug safety of these derivatives for mice.

### 2.6 Biosafety assessment

**FIGURE 8:**
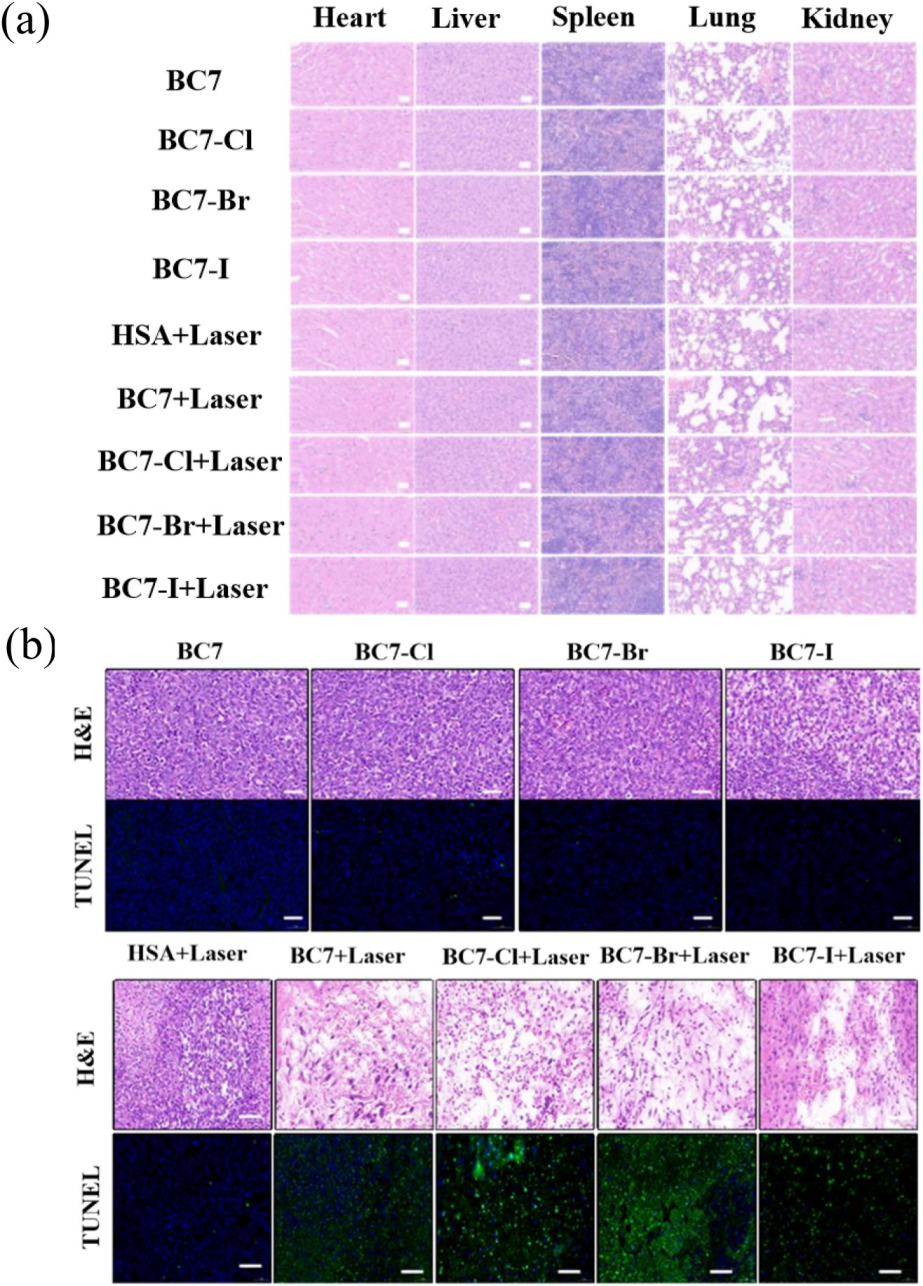
(a) H&E staining of various organs of mice in different treatment groups (bar = 50 μm). (b) TUNEL staining and H&E staining of tumors with different treatments under the no laser irradiation group and laser irradiation group (bar = 50 μm). H&E, hematoxylin‐eosin; TUNEL, terminal transferase‐mediated nick end labeling.

At the end of the treatment cycle, to assess in depth the biosafety of each molecule, we performed humane euthanasia of the mice at the end of the experiment and performed immediate autopsies to remove various tissue organs and tumor tissues. Subsequently, hematoxylin‐eosin (H&E) staining was performed on these tissues, and deoxyribonucleic acid terminal transferase‐mediated nick end labeling (TUNEL) staining was applied to the isolated tumor samples for analysis in order to further explore the necrotic status of the tumor tissues. As shown in Figure 8a, no significant inflammatory response or tissue damage was observed in the main organs of all control and experimental group mice. This indicates that these molecules have precise tumor site targeting and are well metabolized in non‐tumor microenvironments and normal tissues and organ sites, thus verifying that there are no significant histopathological abnormalities under testing conditions. Analysis of H&E staining and TUNEL staining of tumor tissue sections from various groups of mice, Figure 8b shows that large areas of tissue necrosis (i.e., hollow areas presented in the images) were observed in the tumor images of several laser illumination groups, especially the BC7‐Br + Laser group and the BC7‐Cl + Laser group, particularly the BC7‐Br + Laser group, which was consistent with the results of the treatment that demonstrated excellent optical treatment results. In contrast, several groups without laser irradiation showed no obvious signs of tumor damage in their images.

## 3 CONCLUSION

Based on the above analysis, we have chemically modified the molecule of BC7, a near‐infrared cyanine dye, with halogen functional groups. Through a series of well‐designed experimental optimizations, the synthetic process of the target compounds was successfully simplified, and the expected derivatives were prepared. First, a series of in vitro characterization experiments verified that the series of molecular derivatives possessed the dual functions of both PTT and PDT. The chemically modified derivatives showed significant enhancement in stability, warming efficiency, and ROS generation compared to the original BC7 molecules. Subsequently, a solution of the molecule was injected into a tumor‐bearing mouse model via the tail vein, and in vivo imaging confirmed that the modified molecule had excellent tumor targeting properties, and its targeting speed was superior to that of the unmodified BC7 molecule. Further in vivo tumor optical treatment experiments showed that tumor growth was significantly inhibited in the BC7‐Br + Laser group of hormonal mice. Even in the BC7‐Cl + Laser group and BC7‐I + Laser group, the therapeutic effect was significantly better than that of the treatment group using only BC7 in combination with laser irradiation. This work provides theoretical guidance and experimental basis for the development of material systems with dual functions of PTT and PDT.

## CONFLICT OF INTEREST STATEMENT

The authors declare no conflicts of interest.

## ETHICS STATEMENT

All animal procedures were carried out under the guidelines set by the Institutional Animal Care and Use Committee of Shandong province, and the overall project protocols were approved by the Animal Ethics Committee of Linyi University.

## Supporting information

Supporting Information S1

## Data Availability

The data that support the findings of this study are available in Supporting Information of this article.
