# Supplementary material for: Photostabilized halocyanine for near‐infrared excited bimodal photothermal synergism and tumor‐targeted therapy
Source: Smart Mol. 2026 Aug 12:e70088. Online ahead of print. doi: 10.1002/smo2.70088 (PMC13469511; doi:10.1002/smo2.70088)
Supplement: Supplementary file 1 [file SMO2-9999-0-s001.docx]

Supporting Information

Photostabilized Halocyanine for Near-infrared Excited Bimodal Photothermal Synergism and Tumor-targeted Therapy

Zongjin Qu ^a #^, Yurong Guo ^b #^, Cuixia Yao ^c *^, Can Wang ^a^, Zhen Wang ^a^, Jiali Li ^b^, Zhao Wang ^b^, Yingying Jing ^b^, Yen Leng Pak ^b^, Xing Gao ^b^, Shusheng Zhang ^a *^

^a^ Shandong Provincial Key Laboratory of Tumor Imaging Equipment Development and Diagnosis & Treatment Integration Technology, Linyi University, Linyi 276000, P.R. China. E-mail: shushzhang@126.com.

^b^ College of Biological and Chemical Engineering, Qilu Institute of Technology, Jinan 250200, P.R. China.

^c^ College of Chemistry and Chemical Engineering, Linyi University, Linyi 276000, P.R. China. E-mail: yaocuixia@163.com.

^#^These authors contributed equally.

**Scheme S1**. Synthetic route of halogenated compounds.

# 1. Synthesis of Halogenated Derivative Molecules of BC7

(1) Synthesis of compound 1:

The synthesis route of compound 1 is shown in Figure S1. Add KI to a dry round-bottom flask equipped with a magnetic stirrer and let it cool thoroughly. Add substrate A under nitrogen protection B, then add ultra-dry CH3CN to the system and reflux at 110°C for 48 hours. After the reaction is complete, excess potassium iodide is removed by filtering through diatomaceous earth, the solvent is spin dried, PE is added, stirred thoroughly, and washed repeatedly several times. After washing with PE, PE/EA (10:1) is repeatedly washed (>5), and pure dark red powder is obtained after washing. (Taking the synthesis of BC7-Bras as an example: the yield of this step is 40%).

(2) Synthesis of compound 2:

The synthesis route of compound 2 is shown in Figure S1. Add substrates A and B to a dry round-bottom flask equipped with a magnetic stirrer, and then let it cool thoroughly. After dissolving in the prepared solvent, the reaction was carried out at 110°C for 12 hours, and the reaction system changed from red to green over time. After the reaction is complete, thoroughly spin dry the solvent in the system, purify it through a silica gel column, first remove small polar impurities with DCM, and then pass the dark green solid product through DCM/MeOH (50/1). After obtaining the solid product, wash it thoroughly multiple times with PE/EA (10/1). After washing, a pure dark red solid product was obtained. (The yield of (BC7-Br is 69%, the yield of (BC7-F is 63%, the yield of BC7-Cl is 73%, the yield of BC7-I is 70%.)

The nuclear magnetic resonance spectrum of compound BC7-F. HRMS（ESI）: calcd. m/z of C_36_H_42_ClF_2_N_2_^+^ [M]^+^ 575.3000; found, 575.9223. ^1^HNMR (300 MHz, CDCl_3_) δ 8.24 (d, J = 14.1 Hz, 2H), 7.21 – 7.17 (m, 2H), 7.06 – 7.00 (m, 4H), 6.01 (d, J = 14.1 Hz, 2H), 4.10 (t, J = 6.8 Hz, 4H), 2.64 – 2.61 (m, 4H), 1.89 – 1.81 (m, 6H), 1.65 (s, 12H), 0.99 (t, J = 7.3 Hz, 6H) ppm.^13^C NMR (75 MHz, CDCl3) δ 172.3, 160.1 (d, 1JC−F= 244.4 Hz), 150.5, 144.1, 142.9 (d, 3JC−F= 8.1 Hz), 138.3, 127.2, 115.6 (d, 2JC−F= 24.1 Hz), 112.3 (d, 3JC−F= 8.5 Hz), 110.2 (d, 2JC−F= 24.9 Hz),101.3, 77.5, 49.5, 46.7, 28.1, 26.6, 20.8,11.7 ppm. ^19^FNMR (282 MHz, CDCl_3_) δ − 115.92 ppm.

NMR spectra of compound BC7-Cl HRMS（ESI）: calcd. m/z of C_36_H_42_^35^Cl_3_N_2_^+^ [M]^+^ 607.2408; found, 607.2398. ^1^HNMR (300 MHz, DMSO) δ 8.24 (d, J= 14.0 Hz, 2H), 7.82 (s, 2H), 7.53 – 7.50 (m, 4H), 6.35 (d, J = 14.0 Hz, 2H), 4.20 (s, 4H), 2.72 (s, 4H), 1.84 – 1.68 (m, 18H), 0.96 (t, J = 7.1 Hz, 6H) ppm. ^13^CNMR (100 MHz, DMSO) δ 177.4, 153.6, 148.3, 146.4, 134.7, 134.0, 133.8, 132.1, 128.3, 118.3, 107.3, 54.4, 50.5, 32.5, 32.4, 31.1, 25.7, 16.3 ppm.

NMR spectra of compound BC7-Br. HRMS (ESI): calcd. m/z of C_36_H_42_^79^Br_2_^35^ClN_2_^+^ [M]^+^695.1398; found, 695.1389. ^1^HNMR (400 MHz, DMSO) δ 8.27 – 8.17 (m, 2H), 7.97 – 7.96 (m, 2H), 7.63 – 7.61 (m, 2H), 7.49 – 7.47 (m, 2H), 6.37 (d, J= 14.1 Hz, 2H), 4.22 (s, 4H), 2.73 (s, 4H), 1.85 – 1.69 (m, 18H), 0.97 (t, J = 7.3 Hz, 6H) ppm. ^13^CNMR (100 MHz, DMSO) δ 172.4, 148.8, 143.8, 143.6, 142.0, 131.8, 127.4, 126.3, 118.0, 114.0, 102.5, 49.6, 45.8, 27.8, 27.7, 26.4, 20.9, 11.6 ppm.

NMR spectra of compound BC7-I. HRMS (ESI): calcd. m/z of C_36_H_42_^35^ClI_2_N_2_^+^ [M]^+^ 791.1120; found, 791.1112. ^1^HNMR (300 MHz, DMSO) δ 8.25 (d, J= 14.0 Hz, 2H), 8.06 (s, 2H), 7.77 (d, J = 8.2 Hz, 2H), 7.34 (d, J = 8.3 Hz, 2H), 6.35 (d, J = 14.0 Hz, 2H), 4.20 (s, 4H), 2.73 (s, 4H), 1.80 – 1.68 (m, 18H), 0.96 (t, J = 7.2 Hz, 6H) ppm. ^13^CNMR (100 MHz, DMSO) δ 172.1, 148.8, 143.9, 143.5, 142.5, 137.6, 131.7, 127.4, 114.3, 102.4, 90.3, 55.4, 49.5, 45.7, 27.8, 26.4, 20.9, 11.5 ppm.

# 2. Optical Characterization

Accurately weighed BC7-F, BC7-Cl, BC7-Br, BC7-I, and commercially available BC7 molecular powders were dissolved in DMSO solution to form a masterbatch (concentration of 10 mmol/L), and then the masterbatch was stored in a cool place protected from light. Each time they were used, they were diluted to the concentration to be measured, and the absorption and fluorescence emission spectra were determined. The absorption spectra were tested in dimethyl sulfoxide (DMSO) solvent, and the scanning range was selected to be 300-900 nm. The emission spectra of the four derivatives and the BC7 molecules were tested in DMSO solution using an F-4600 fluorescence spectrophotometer. The excitation wavelength (*E*x) was set to 740 nm, the scanning wavelength range 750-900 nm, and the slit width 5 nm.

3. Photothermal conversion efficiency

In order to supplement the photothermal conversion efficiency of each photosensitizer, we use the standard photothermal experimental model for quantitative calculation. Under the approximate condition of neglecting the background heat loss of the container and solvent, the photothermal conversion efficiency can be calculated by the following formula:

η≈

Where *hS* is the heat dissipation coefficient of the system, which can be derived from the thermal time constant τ (*hS*=mC/τ); I is the incident laser power; A_λ_ is the absorbance of photosensitizer at 808 nm.

According to the heat transfer time constant of the photosensitizer system and the photothermal conversion efficiency formula of the photosensitizer, we obtained that the photothermal conversion efficiency of different photosensitizers were 30.1% for BC7, 36.7% for BC7-Cl, 34.2% for BC7-Br and 31.5% for BC7-I.

4. **Computational Calculations**

The ground state molecular structures were optimized using Gaussian 16 with the B3LYP function and def2svp basis set. ^[1]^ The solvent used was dimethyl sulfoxide to maintain consistency with experimental conditions according to the previous study, and its effects were included using the SMD module. The singlet emission energies were calculated using TD-DFT with the B3LYP function and def2svp basis set in Gaussian 16. The triplet emission energies were calculated using TDA-DFT with triplet states at the same levels as the other calculations. The solvent used was dimethyl sulfoxide, and its effects were included using the SMD module. In addition, the spin-orbit coupling matrix elements were obtained using ORCA software under the same levels and conditions as the Gaussian calculations.^[2]^ In order to investigate the electronic behavior of photosensitizer molecules, we used the Multiwfn software to output the hole-electron CUB file.^[3]^ CUB files containing hole-electron orbital information were visualized using the software VMD.

# 5. In vitro Photothermal Performance Characterization

Various molecular compounds were accurately weighed and dissolved in dimethyl sulfoxide (DMSO) to form a masterbatch at a concentration of 10 mmol/L. The masterbatch was stored for a short period of time under light-protected conditions.

(1) **Power Dependence test**

The mother liquor of each molecule was diluted into several milliliters of DMSO solution of 8 μmol/L concentration, and 1 mL of the solution was pipetted sequentially into a small 1.5 mL centrifuge tube for testing. An 808 nm laser with different power densities (0.308 W/cm^2^, 0.697 W/cm^2^, 0.861 W/cm^2^, 1.295 W/cm^2^, 1.839 W/cm^2^) was used to irradiate the fixed centrifuge tubes containing the solutions to be tested, and a hand-held near-infrared thermography (NIRT) camera was used for real-time monitoring of the warming process.

(2) **Photothermal Stability Test**

The mother liquor was diluted into 10 μmol/L DMSO solutions of BC7, BC7-F, BC7-Cl, BC7-Br, and BC7-I, respectively, and the fluorescence emission was measured after each irradiation by 808 nm laser irradiation (at 5-minute intervals, and then at 1-minute intervals after 10 min of irradiation) with the power density set at 0.861 W/cm2. The fluorescence emission was measured after each irradiation.

# 6. Evaluation of Active Oxygen Generation Capacity in Solution

Based on domestic and international research progress, as well as previous studies by our research group, we conducted a preliminary study on photodynamic therapy (PDT) of BC7 and each derivative molecule of BC7 to test their reactive oxygen species-generating capacity within the solution. The five molecules were mixed with DPBF (a single-linear oxygen-indicating fluorescent probe) solution in a certain ratio: the mother liquor of each molecule was diluted into 5 μmol/L test solution, and 200 μmol/L DPBF solution was prepared, and then they were mixed in the ratio of 1:1 (the molecules and the DPBF were used in the DMSO solution as solvents) Immediately after irradiation with 808 nm laser, the absorbance change of each solution was measured by UV-visible spectrophotometer with 30 s/time of irradiation, and three power densities of 0.308 W/cm^2^, 0.861 W/cm^2^, and 1.295 W/cm^2^ were selected for the parallel experimental study.

# 7. Cell Culture

Drugs and reagents required for cell culture: complete medium: DMEM medium, 1% penicillin-streptomycin, and 10% fetal bovine serum (FBS). The MCF-7, HeLa, 4T1, and L02 cells used were cultured in a 37℃ constant temperature incubator, in which 95% CO_2_ gas concentration was maintained.

# 8. Cytotoxicity Assay

Cells with good growth status were inoculated into 96-well plates, and the same cell density was maintained in each well. And incubated in a constant temperature and humidity incubator overnight, waiting for the cells to grow to a suitable density. The old culture solution was aspirated and replaced with molecular solutions configured with different concentrations of culture solution, and the cells continued to be co-incubated with the cells for a certain period of time to replace the cck-8 culture solution, and absorbance was detected with an enzyme marker around 25 min. The concentration gradients of 0, 0.5, 1, 2.5, 5, and 10 μmol/L were set up, and the toxicity tests were carried out with and without light conditions, respectively.

Select the cells with good growth status, collect the cell suspension after treatment, take 100 μL of cell suspension and spread it in a 96-well plate (about 8000 cells/well cell density), then transfer the well plate to the cell culture incubator and incubate for 12 h overnight. After the cells were well adhered to the wall and the density was suitable, the old culture solution was replaced by the synthesized compounds and BC7molecules with a set concentration gradient, and the solution was aspirated out of the wells one by one for a certain period of time, and 100 μL of CCK-8 solution (10% CCK-8 + 90% medium) was re-added in the same order. The 96-well plates under the same treatment conditions were subjected to the application of an 808 nm near-infrared laser and no laser treatment, i.e., to test the phototoxicity and dark toxicity of the cells, respectively. Then the treated 96-well plates were re-incubated in the incubator for 25-60 min, and then the plates were taken out, and the absorbance was measured by an enzyme marker.

# 9. Evaluation of Intracellular Reactive Oxygen Species Production Capacity

Cells with good growth status were inoculated in confocal dishes and incubated overnight. After the cells were observed to be firmly attached to the wall and had a good morphology in the microscope, the old culture medium was replaced with the medium solution of BC7-F / BC7-Cl / BC7-Br / BC7-I and BC7, and the incubation was continued for 5-6 h. After the PBS was washed for 2-3 times, the DCFH-DA solution configured with pure DMEM was added and incubated with the cells for 25 min, and then the DCFH-DA solution, which had not entered the cells, was washed out with PBS and reintroduced into the pure DMEM. Then PBS was washed off the DCFH-DA that did not enter the cells, and pure DMEM was added again. 808 nm laser irradiation was performed for 5 min, and the output of intracellular reactive oxygen species was observed by confocal microscopy.

# 10. Establishment of Mouse Tumor Model

The mice used in the experiments were SPF-grade Balb/c female nude mice, which were about five weeks old at the time of purchase, and were purchased from Beijing Viton Lihua Laboratory Animal Technology Co. The mice were weighed and divided into cages of 5 mice each. The temperature and humidity in the room were controlled to be the most suitable environment for survival, and the feed, water source, and bedding were regularly replenished and replaced. The mice were kept for 7-10 days for experimental treatment, during which time they should be observed at any time and weighed at regular intervals. During the same period, the tumor cells (4T1) were cultured, and the mice were inoculated with tumors when their body weight grew to about 18 g. The mice were then inoculated with tumors. After the 4T1 cells with good growth status were digested with trypsin, the cell suspension was collected and dispersed into PBS (Phosphate Buffered Salt Solution), and the cells were counted. Nude mice eligible for inoculation were sterilized with alcohol, and 100 μL of cell suspension was injected subcutaneously into the right hind leg. Records of inoculation were made, and the mice were continued to be kept for observation of tumor growth at any time.

# 11. Tumor in Vivo Imaging

Several tumor-bearing mice with similar tumor volumes were selected and injected with PBS solution doped with several molecules (containing human serum albumin) into their tail veins. Each mouse was anesthetized and placed in an in vivo imager for in vivo imaging as well.

# 12. In Vivo Anti-tumor Assay

Nude mice inoculated with tumor cells will continue to be housed for observation. Mice will be weighed daily, and tumors will be observed. Tumor prototypes can be observed about 3 days after inoculation, after which tumor measurements are taken and recorded daily (treatment starts when the tumor volume is 100 mm³). The tumor-bearing mice were divided into nine experimental groups of five mice each: (1) BC7; (2) BC7-Cl; (3) BC7-Br; (4) BC7-I; (5) HAS + Laser group; (6) BC7+ Laser group; (7) BC7-Cl + Laser group; (8) BC7-Br + Laser group; (9) BC7-I + Laser group. Tumor volume calculation formula: V = 1/2 × length × width × width.

# 13. Biosafety Assessment

To further assess the biosafety of each molecule and for pathological testing, mice were subjected to humane execution at the end of the treatment cycle and immediately dissected to remove various tissues and organs (including heart, liver, spleen, lungs, and kidneys) and excise tumors. Then they were sequentially sectioned, fixed, dehydrated, paraffin-embedded, and stained with H&E. The collected tumors were also analyzed by TUNEL staining to further study the necrosis of the tumor tissues.

**Figure S1**. ^1^H NMR spectrum of compound BC7-F.

**Figure S2**. ^13^C NMR spectrum of compound BC7-F.

**Figure S3**. High-resolution mass spectrum of BC7-F.

**Figure S4**. ^19^F NMR spectrum of compound BC7-F.

**Figure S5.** High-resolution mass spectrum of BC7-F.

**Figure S6**. ^1^H NMR spectrum of compound BC7-Cl.

**Figure S7**. ^13^C NMR spectrum of compound BC7-Cl.

**Figure S8**. High-resolution mass spectrum of BC7-Cl.

**Figure S9**. ^1^H NMR spectrum of compound BC7-Br.

**Figure S10**. ^13^C NMR spectrum of compound BC7-Br.

**Figure S11**. High-resolution mass spectrum of BC7-Br.

**Figure S12**. ^1^H NMR spectrum of compound BC7-I.

**Figure S13**. ^13^C NMR spectrum of compound BC7-I.

**Figure S14**. High-resolution mass spectrum of BC7-I.

**Figure S15** (a-d) temperature rise (ΔT) of photosensitizer irradiated by 808 nm laser; Cooling diagram after turning off the laser after 600 s of infrared radiation. (e-h) The heat transfer time constant of the system is obtained by fitting the linear time data Vs − ln (θ) in the cooling phase, where θ is the driving force of temperature.

**Figure S16**. The most stable ground state structures of cyanine derivatives with different halogen substitutes. (Grey: carbon element; White: hydrogen element; Blue: nitrogen element; Green: chlorine element; Brown red: bromine element; Purple: iodine element)

**Figure S17**. Theoretical absorption and emission spectra of different BC-X compounds in DMSO solvent.

**Figure S18**. The optimal structure of the BC7 molecule, the highest occupied molecular orbital (HOMO) / lowest unoccupied molecular orbital (LUMO) surface of the BC7 molecule, as well as the range of excited state energy and spin-orbit coupling constant.

**Figure S19**. Phototoxicity of BC7-Br and L02 cells at different concentrations.

References

[1] M. E. Frisch, G. W., Trucks, H. B., Schlegel, G. E., Scuseria, M., Robb, J. R., Cheeseman, G., Scalmani, V. P. G. A., Barone, G. A., Petersson, H. J. R. A. Nakatsuji, and X., Li, Gaussian 16, Revision B.01, DOI: 2016. 10.1063/1.4943093.

[2] Neese F.; Software update: The ORCA program system—Version 5.0. Wiley *Interdisciplinary Reviews-Computational Molecular Science* **2022**; *12*, e1606. DOI: ‌10.1002/wcms.1606.‌

[3] T., Lu, F. Chen, Multiwfn: A multifunctional wavefunction analyzer, *Journal of Computational Chemistry*, **2012**, *33*, 580-592. DOI: 10.1002/jcc.22885.
